# Magnetic Aerogels for Room-Temperature Catalytic Production of Bis(indolyl)methane Derivatives

**DOI:** 10.3390/molecules29102223

**Published:** 2024-05-09

**Authors:** Nicola Melis, Danilo Loche, Swapneel V. Thakkar, Maria Giorgia Cutrufello, Maria Franca Sini, Gianmarco Sedda, Luca Pilia, Angelo Frongia, Maria Francesca Casula

**Affiliations:** 1 Department of Mechanical, Chemical and Materials Engineering, University of Cagliari, 09123 Cagliari, Italy; 2 Nanostructures & Biotech Laboratory, Biological and Environmental Science and Engineering (BESE) Division, King Abdullah University of Science and Technology, Thuwal 23955-6900, Saudi Arabia; 3 Department of Chemical and Geological Sciences, University of Cagliari, 09042 Monserrato, Italy

**Keywords:** aerogel, ferrite, nanocomposite, catalyst, organic synthesis, bis(indolyl)methanes

## Abstract

The potential of aerogels as catalysts for the synthesis of a relevant class of bis-heterocyclic compounds such as bis(indolyl)methanes was investigated. In particular, the studied catalyst was a nanocomposite aerogel based on nanocrystalline nickel ferrite (NiFe_2_O_4_) dispersed on amorphous porous silica aerogel obtained by two-step sol–gel synthesis followed by gel drying under supercritical conditions and calcination treatments. It was found that the NiFe_2_O_4_/SiO_2_ aerogel is an active catalyst for the selected reaction, enabling high conversions at room temperature, and it proved to be active for three repeated runs. The catalytic activity can be ascribed to both the textural and acidic features of the silica matrix and of the nanocrystalline ferrite. In addition, ferrite nanocrystals provide functionality for magnetic recovery of the catalyst from the crude mixture, enabling time-effective separation from the reaction environment. Evidence of the retention of species involved in the reaction into the catalyst is also pointed out, likely due to the porosity of the aerogel together with the affinity of some species towards the silica matrix. Our work contributes to the study of aerogels as catalysts for organic reactions by demonstrating their potential as well as limitations for the room-temperature synthesis of bis(indolyl)methanes.

## 1. Introduction

Unique features of aerogel materials such as their extremely high and open porosity, the controlled and tunable composition and purity, as well as the ability to stabilize highly dispersed active metal-based nanocrystals, make them ideal candidates as catalysts [1,2,3].

The use of aerogels in heterogeneous catalysis dates back to pioneering work by Pajonk, where simple and mixed oxide aerogels were applied to selective oxidation, hydrogenation, and reduction reactions, taking advantage of the high thermal and time-on-stream stability of oxide aerogels [4,5,6]. Since then, aerogels have been used as catalysts and supports for catalytic active phases for a broad range of processes, including biocatalysis [7].

Current trends in silica-based materials, which rank among the most investigated aerogels, have been recently reviewed and include nanofiber-reinforced and silica-hybridized cellulose for targeted applications such as thermal insulation [8,9,10]. In particular, advances in the production of aerogel composites have enabled the preparation of catalysts where the catalytically active nanocrystalline phase is finely dispersed over a highly porous silica matrix. Such nanocomposite aerogels have been demonstrated to be active as heterogeneous catalysts for a wide range of gas-phase reactions relevant for energy and technological applications, such as the Fischer–Tropsch process, the Catalytic Chemical Vapor Deposition production of carbon nanotubes, and the Water–Gas Shift reaction [11,12,13,14]. Together with the peculiar textural features of aerogels, which may confer improved catalytic performance as compared to the corresponding xerogels, a major advantage associated with the dispersion of catalytically active nanocrystals into highly porous aerogel matrices is that sintering is prevented during operating conditions, retaining the catalytic performance [12,15].

Molins and coworkers have investigated the use of silica-based and carbon-based nanocomposite aerogels as catalysts for organic reactions such as Mizoroki–Heck coupling and Biginelli condensation reactions [16,17]. This innovative approach offers the opportunity to explore the potential of novel aerogel-based catalysts as an alternative to conventional homogeneous catalysts used for organic processes. Among the advantages of using a solid catalyst, major aspects relate to the ease of separation of the catalyst from the reaction mixture, enabling therefore a more cost- and time-effective process and opening the way for catalyst reuse. In particular, together with filtration, magnetic separation of the catalyst from the reaction mixture can be easily performed by using magnetic solid catalysts. In addition, by taking advantage of the fine tuning enabled by sol–gel chemistry, the catalyst performance can be optimized by combining optimized features of the matrix and supported phase, as demonstrated by the design of alkylation catalysts based on metal halide supported on acidic silica-alumina aerogel matrix [18].

In this work, we investigate, to the best of our knowledge for the first time, the use of nanocomposite magnetic aerogels as catalysts for the synthesis of bis(indolyl)methanes starting from indole and aromatic aldehydes.

Bis(indolyl)methanes (BIMs) rank among the natural bis-heterocyclic compounds whose relevance is related to their broad occurrence in terrestrial and marine sources as well as to their biological role as metabolites [19]. Synthetic BIMs represent relevant organic intermediates for the pharmaceutical and chemical industry, with potential areas of applications ranging from colorimetric ion detection to dietary supplements [20]. Recently, BIMs have attracted great interest due to their prospective pharmacological properties such as antiproliferative and anti-cancer activity [21,22]. 

**Figure 1:**
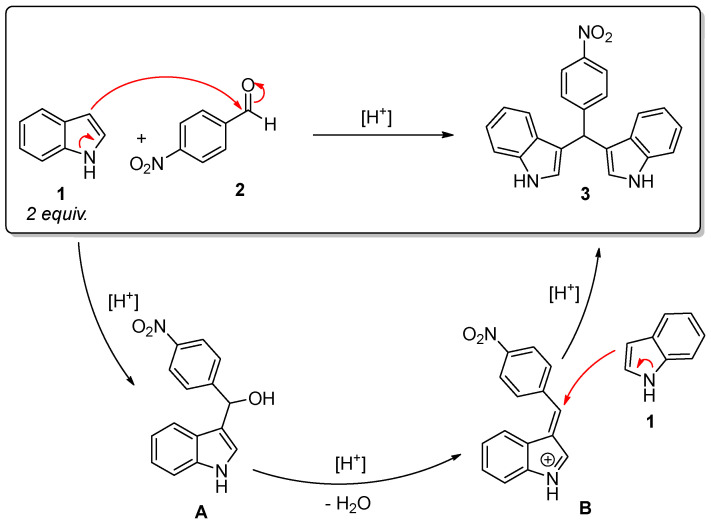
Mechanism for the formation of bis(indolyl)methane **3** from indole **1** and 4-nitrobenzaldehyde **2**. Red arrows suggest possible electron rearrangements.

The wide range of applications in the synthesis of organic derivatives, as well as potential applications in the detection, sensing, biological, and pharmaceutical fields, have motivated the research on synthetic approaches to BIMs. BIMs are usually obtained by acid-catalyzed electrophilic substitution reaction of indole with a carbonyl compound such as an aldehyde or a ketone. The overall process implies a reaction between indole **1** and an aromatic aldehyde **2** leading to the formation of the corresponding alcohol **A** and, upon dehydration, of the corresponding azafulven intermediate **B**, as shown in Figure 1. This intermediate undergoes the subsequent addition of a second indole molecule to yield the final BIM derivative. In this classic approach, both Lewis and Brønsted acids have been proved to act as effective catalysts of the reaction.

Alternative strategies include synthetic protocols starting from intermediate **A** or **B**. In particular, based on such a methodology, the synthesis of asymmetric BIMs was reported by Bergman [23]. Moreover, BIMs have been produced through photochemical-induced methods [24,25,26], and ultrasound and microwave irradiation [27,28], with the aim to establish green and health-caring approaches. 

Nevertheless, the standard method remains the most straightforward and versatile approach for access to BIM derivatives. In fact, the reaction has been extensively studied using a wide variety of Brønsted and Lewis acids in solution and several solids with acid behaviour for heterogeneous catalysis. Mineral acids (such as HCl, HBr, or H_2_SO_4_) and organic acids (such as formic or acetic acids [29,30,31,32], sulfamic acid [33,34,35,36], or p-Toluenesulfonic acid [37]) have been successfully employed as catalysts in this method. Furthermore, tests of a wide selection of Lewis acids (such as chlorides, oxides, and other salts of transition metals such as Fe, Cu [38,39,40,41,42], Zn [43,44], Al [45,46], Ti [47,48,49], and Zr [50], along with different examples of rare earth salts) have been reported in the literature. Among other examples, Wang and co-workers studied with an extensive screening the effect of the nature of the Lewis acids used as catalytic species in the reaction between indole and benzaldehyde [51].

As an alternative to the synthesis under homogeneous catalysis, different approaches based on heterogeneous catalysis have been proposed, including those based on HY and ZnY zeolites [52,53,54], polystyrene-based resins [55], amberlyst [56,57,58], PEG-supported sulfonic acid [59], lanthanide resins [60], and montmorillonite clay K-10 catalysts [61].

Nanocrystalline catalysts such as semiconducting CdS nanorods have also been proposed for the synthesis of bis(indolyl)methanes in different media at reflux temperature [62], opening the way to greener approaches such as solvent-free reaction at 80 °C mediated by magnetically recoverable γ-Fe_2_O_3_ nanocatalysts [63]. Indeed, the advantages of a magnetic functionality in catalyst separation, recovery, and reuse has been reported for diverse organic reactions, ranging from the synthesis of heterocycle building blocks (spiropyrans) to the reduction of environmental contaminants (dyes, drugs) [64,65,66]. Nanocrystals, however, may suffer from aggregation under reaction conditions, and novel nanocomposite materials based on the immobilization of catalytically active phases on supports such as carbon nanotubes and graphene oxide aerogels have been proposed [67,68].

In this work, we made use of nanocomposite aerogels which combine the features of iron-based magnetic nanocrystals and of highly porous silica to address the catalytic synthesis of 3,3′-((4-nitrophenyl)methylene)bis(1*H*-indole). The investigated catalysts were highly porous NiFe_2_O_4_/SiO_2_ aerogel nanocomposites containing ferrite nanocrystal with narrow size distribution around 10 nm. The investigated materials were effective catalysts for the synthesis of BIMs at room temperature, demonstrating the potential of nanocomposite aerogels for the catalytic production of relevant heterocyclic compounds.

## 2. Results

### 2.1. Catalyst Preparation and Characterization

**Figure 2:**
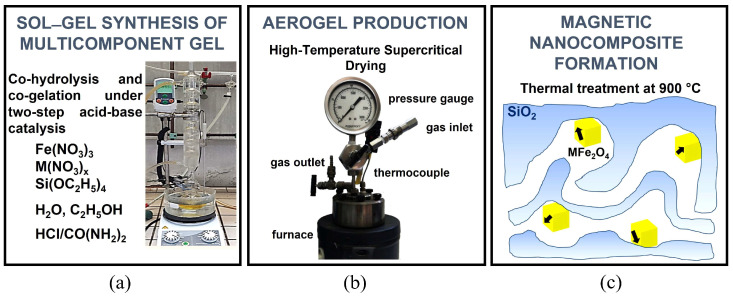
Schematic of the production procedure of the aerogel catalysts: (**a**) sol–gel synthesis of the multicomponent gel by co-hydrolysis and co-gelation of the metal and silica precursors; (**b**) aerogel production by high-temperature supercritical drying of the multicomponent gel; (**c**) thermal treatments to promote the formation of magnetic NiFe_2_O_4_/SiO_2_ nanocomposite aerogel catalysts.

The aerogel catalysts were prepared by a sol–gel procedure followed by supercritical drying and thermal treatments, as depicted in Figure 2. The synthetic approach is based on a protocol established in our laboratories which enables the production of multicomponent silica-based gels by a two-step acid-base urea-mediated catalysis of metal and silica precursors. Aerogels are produced by a high-temperature supercritical drying, and finally the MFe_2_O_4_/SiO_2_ nanocomposite aerogels are obtained by thermal treatment to promote the formation of the ferrite nanocrystals dispersed on porous amorphous silica. The formation of the desired nanocrystalline phase for this work, NiFe_2_O_4_, was assessed by X-ray powder diffraction (see Supplementary Materials, Figure S1) which showed the occurrence of the ferrite spinel phase superimposed to the broad halo due to amorphous silica. XRD peak broadening indicates an average nanocrystalline domain size around 8 nm for the NiFe_2_O_4_ phase. In the multicomponent aerogels as obtained right after supercritical drying, the dispersed metals occur in poorly ordered nanophases which are non-magnetic or poorly magnetic, depending on the composition of the composite. On the other hand, upon calcination, highly magnetic nanocrystalline substituted ferrites which readily respond to an external magnet are obtained. Thermal treatments also affect the surface properties of the aerogels. In particular, the aerogels as produced by high-temperature supercritical drying are hydrophobic as surface silanols undergo esterification by ethoxy functional groups during the drying process. When the aerogel is treated in air, the ethoxy groups are removed and, as a consequence of surface silanols, the aerogel becomes hydrophilic [2,69].

**Figure 3:**
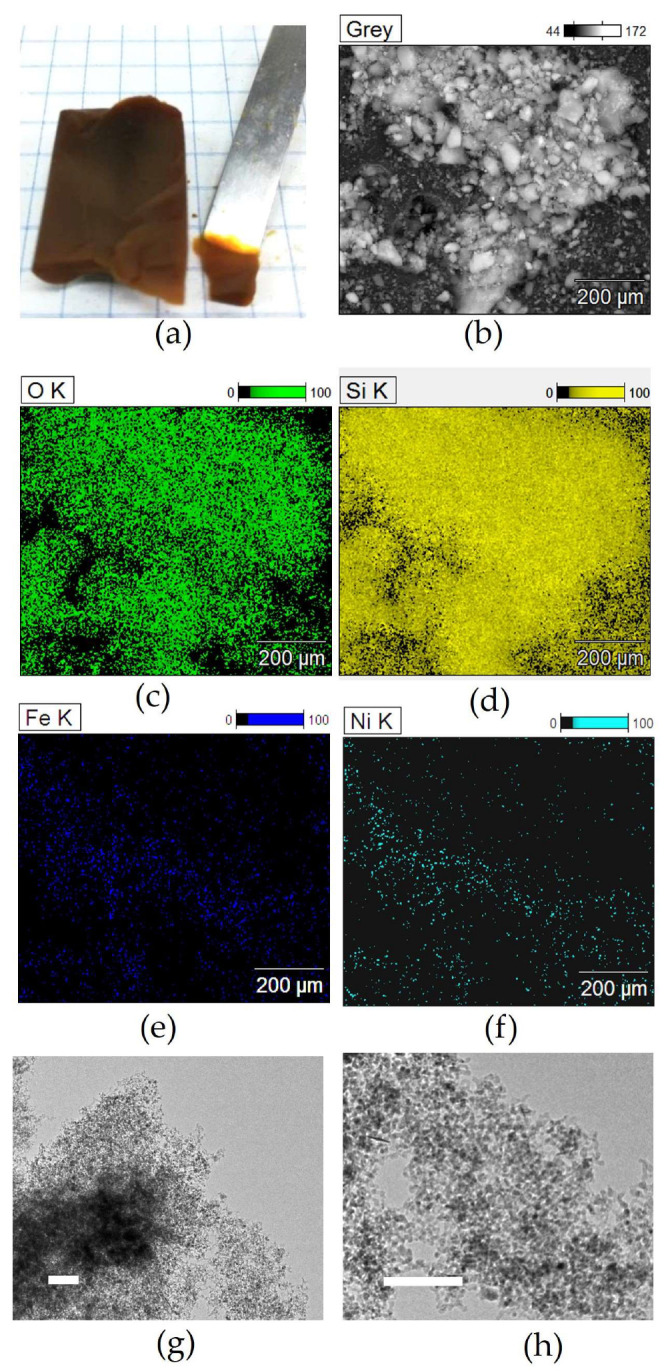
Representative images of the aerogel catalyst at different stages of nanocomposite preparation: (**a**) optical image of a highly porous nickel-containing composite aerogel as obtained after supercritical drying; (**b**) corresponding SEM image and (**c**–**f**) energy-filtered images showing oxygen distribution (**c**), silicon distribution (**d**), Fe distribution (**e**), and nickel distribution (**f**). TEM images (scale bar is 100 nm) of the NiFe_2_O_4_/SiO_2_ aerogel catalyst as obtained by calcination at 900 °C (**g**,**h**).

The nickel-based composite aerogel, as shown in Figure 3a, is extremely lightweight and homogeneous. Scanning electron microscopy and energy-filtered images, acquired in order to assess compositional homogeneity, showed that the distribution of the present elements is quite homogeneous within the aerogel, as demonstrated by representative micrographs shown in Figure 3b–f. It is found that Si and O are relatively more intense as compared to Ni and Fe, as expected based on the gel composition which was adjusted in order to achieve a ferrite loading of 10 wt%, and in particular that Ni and Fe are correlated, suggesting that the two metals are associated in the same phase. Thermal treatment up to 900 °C promotes the formation of the ferrite nanocrystals, which appear as round-shaped darker spots superimposed to the silica matrix under transmission electron microscopy imaging, as shown in Figure 3g,h. TEM images clearly point out that the nanocrystals have an average size around 8 nm in the Ni-CAT and are well dispersed in the silica matrix, as is desirable for prospective use in catalysis. TEM observations (see also Figure S1) indicate that, despite the thermal treatment at 900 °C, and thanks to the very high porosity of the starting aerogel, the NiFe_2_O_4_/SiO_2_ nanocomposite still exhibits the typical texture of aerogels with high and open porosity.

The textural features of the NiFe_2_O_4_/SiO_2_ aerogel catalysts were investigated, together with that of corresponding plain SiO_2_ aerogel, by N_2_ physisorption analysis at 77 K, which indicated (Figure S2) the occurrence of a porous texture dominated by large mesopores. In particular, the isotherm of SiO_2_ can be described as a type IVa isotherm with a narrow H1 hysteresis lying at high relative pressures, suggesting the occurrence of large mesopores with relatively uniform diameter. The nanocomposite aerogel exhibits a similar physisorption isotherm of IIb type, suggesting the occurrence of a very similar texture typical of aerogel structures, associated to interconnected nearly cylindrical mesopores with large sizes [70]. The relevant textural parameters of the aerogel catalysts as obtained by physisorption data analysis are summarized in Table 1, where the porosity of the nanocomposite aerogels can be mainly ascribed to the occurrence of mesopores, while the micropore contribution is negligible. On the other hand, a contribution arising from macropores, which cannot be directly detected by N_2_ physisorption, cannot be ruled out and seems to be suggested by TEM observations.

### 2.2. Catalytic Activity Evaluation

The potential of the NiFe_2_O_4_/SiO_2_ nanocomposite aerogels as acid catalysts for the synthesis of a relevant class of heterocyclic compounds such as BIMs was investigated. In particular, the studied reaction is represented in Table 2 and involves the formation of 3,3′-((4-nitrophenyl)methylene)bis(1*H*-indole) **3** by reaction of two equivalents of indole **1** with 4-nitro-benzaldehyde **2**.

The reaction was carried out, under stirring, over 1 week at room temperature using 5 mol% of NiFe_2_O_4_/SiO_2_ nanocomposite aerogel (Ni-CAT) in CH_2_Cl_2_ and in the presence of plain SiO_2_ (SiO_2_-CAT) as well as without any catalyst. The reaction was monitored by ^1^H NMR analysis of the crude mixture (see Supplementary Materials, Figures S3 and S4) [21,71].

**Figure 4:**
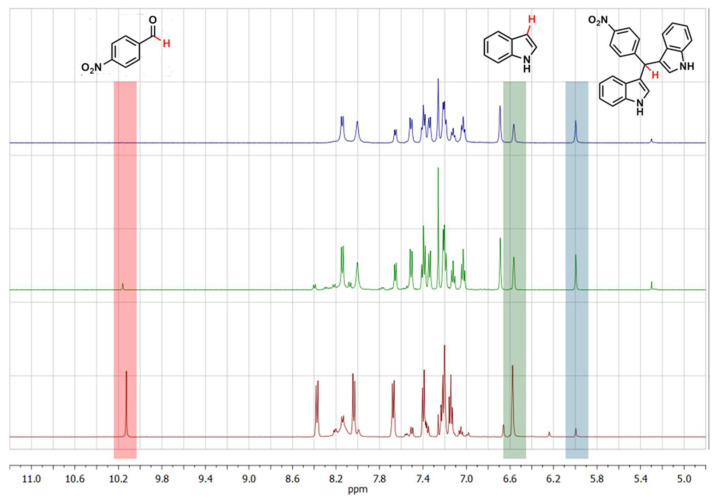
^1^H NMR spectra of the reaction mixture as obtained without catalyst (red bottom curve); with the use of plain SiO_2_ aerogel catalyst (green intermediate curve); and with the use of NiFeO_2_-SiO_2_ aerogel catalyst (top blue curve). Significant spectral ranges with corresponding attribution are included as a guide (catalyst amount: 5 mol %; run time: 1 week; solvent: CH_2_Cl_2_; reaction temperature: RT).

Figure 4 shows a comparison of the reaction mixtures as obtained with the Ni-CAT, and with a SiO_2_-CAT and without catalyst as a reference in a relevant spectral range.

It was found that when the reaction is carried out without the use of any catalyst, after 1 week the desired BIM product **3** represents only 8% of the final reaction mixture. When SiO_2_-CAT is used, a very significant increase in the product formation is observed, up to 60% of the final reaction mixture, while using Ni-CAT leads to 94% of the desired product, together with an almost complete consumption of the aldehyde **2**. The results demonstrate that SiO_2_-CAT is already active towards the investigated reaction, as a result of its high porosity (Table 1) and expected surface features. The occurrence of the nanocrystalline nickel substituted ferrites improves the yield of the product **3** and leads to an almost quantitative consumption of the aldehyde **2**. These results were highly encouraging when taking into account that the reaction was carried out at room temperature. The **1**:**2** ratio in the reaction mixture does not agree with the expected stoichiometry; the observed differences could be due both to the fact that the reaction does not take place in a single step, as described earlier, as well as to interactions of the species involved in the reaction with the porous aerogel matrix, as will be discussed later on.

It should be noted that Ni-CAT offers the advantage of an easy and effective separation procedure of the catalyst from the reaction mixture by the use of an external magnet which selectively separates the catalyst. On the other hand, when the reaction was performed by using SiO_2_-CAT, a filtration process was required to separate and remove the catalyst from the reaction mixture. Screening of the reaction in other solvents, using Ni-CAT, gave considerably poorer yields of **3** (see Table S1). Based on these results, CH_2_Cl_2_ was used for further experiments.

**Figure 5:**
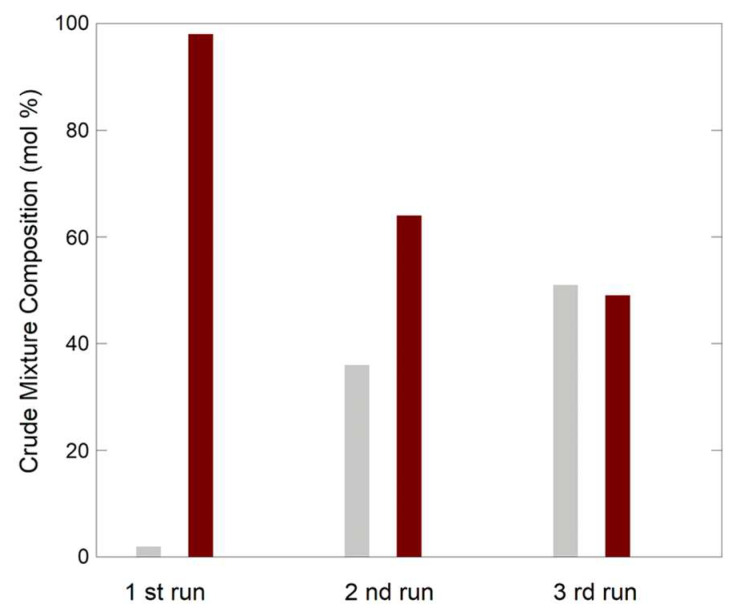
BIMs synthesis catalyzed by Ni-CAT aerogel catalysts (catalyst amount: 5 mol %; run time: 1 week; solvent: CH_2_Cl_2_; reaction temperature: RT): the composition of the resulting reaction mixture is represented as relative amounts of reactant **2** (grey bars) and product **3** (brown bars).

For practical applicability of a heterogeneous catalyst, recyclability is a very important factor. Therefore, we investigated the Ni-CAT aerogel in repeated cycles of reaction. Figure 5 shows that Ni-CAT aerogel, which can be recovered by magnetic separation from the reaction mixture, is still effective in catalyzing BIM synthesis after three repeated runs, although a decreased efficiency is progressively observed.

As a matter of fact, characterization of the aerogels after catalytic runs by scanning and transmission electron microscopy do not provide strong evidence of significant changes in the morphology and microstructure of aerogel after the catalytic runs (see Supplementary Materials, Figure S5-1). However, the XRD pattern of the Ni-CAT after catalysis (Figure S5-2) shows the occurrence of a peak around 2θ ≈ 18° which was not present in the original nanocomposite aerogel. Although the observed pattern does not enable us to identify unambiguously the additional phase present, there are some similar features in the pattern of the BIM product. The same peak was observed when a nanocomposite aerogel catalyst prepared under similar conditions but with different ferrite composition (MnFe_2_O_4_/SiO_2_) was used as a catalyst, supporting the idea that the observed feature is not related to nickel-based phases. In addition, elemental (CHN) analysis of the Ni-CAT aerogel after use suggests the occurrence of C, H, N (11.78% C; 0.48% H; 1.23% N) but there is no match with any of the three species involved.

**Figure 6:**
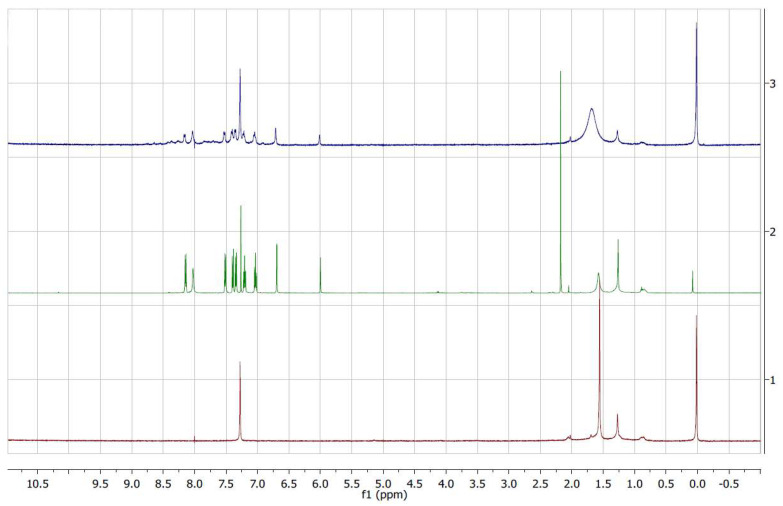
^1^H-NMR spectra of pure BIM (green line), and the extracts after 1 (blue line) and 3 (red line) catalytic runs.

These findings suggest that, although the Ni-CAT aerogel was isolated from the reaction mixture by magnetic separation, and washed repeatedly with CH_2_Cl_2_, it is likely that some of the species involved in the reaction were trapped in the aerogel. The Ni-CAT aerogel after one catalytic run and after three catalytic runs was therefore treated with hot methanol and the corresponding extract was analyzed by ^1^H NMR. Only compound **3** was observed and no traces of the corresponding starting materials **1** and **2** were detected, as illustrated in Figure 6. It should be pointed out, however, that the investigated reaction takes place with the formation of a transient polar intermediate such as **A** (see Figure 1); hence, its interaction with the porous support during the reaction, with subsequent trapping, cannot be completely ruled out. The extract from a catalyst used for three repeated runs does not show significant signals; hence, it is likely that the lower catalyst activity is associated with lower interaction and trapping of the reaction species. Overall, these observations (^1^H NMR data together with insights from characterization of the catalyst after use) are consistent with the hypothesis that some reaction species (compound **3** and probably intermediate **A**) are trapped within the highly porous aerogel.

## 3. Materials and Methods

### 3.1. Materials

Chemicals were purchased from the following providers and used without further purification. BIM synthesis: Indole (99+%, Aldrich, St. Louis, MO, USA); 4-Nitrobenzaldehyde (>99%, Aldrich); Dichloromethane (>99%, Aldrich); Acetone (>99.5%, Honeywell, Charlotte, NC, USA); Acetonitrile (>99.5%, puriss. p.a., ACS reagent, reag. Ph. Eur., Aldrich); Chloroform (99.8%, ACS, Alfa Aesar, Haverhill, MA, USA); Diethyl ether (RPE-For analysis-ACS, Carlo Erba Reagents, Cornaredo, Italy); Ethanol (96–97.2%, Honeywell); Ethyl Acetate (>99.7% Chromasolv, Sigma-Aldrich, St. Louis, MO, USA); 2-methyltetrahydrofuran (99%, Alfa Aesar); Toluene (>99.5% ACS Reagent, Sigma-Aldrich).

Aerogel catalyst synthesis: tetraethoxysilane ((Si(OC_2_H_5_)_4_, Aldrich 98%, TEOS); Urea ((NH_2_CONH_2_), Sigma-Aldrich (99.0–100.5%); Absolute Ethanol (CH_3_CH_2_OH, Carlo Erba); Nitric Acid (HNO_3_, Carlo Erba); Iron (III) nitrate nonahydrate (Fe(NO_3_)_3_·9H_2_O, Sigma-Aldrich 98%); Manganese (II) nitrate hexahydrate Mn(NO_3_)_2_·6H_2_O, Sigma-Aldrich, ≥98%); Nickel (II) nitrate hexahydrate Ni(NO_3_)_2_·6H_2_O, Sigma-Aldrich, ≥97%).

### 3.2. Synthesis of Aerogel Catalysts

The aerogels were produced by a previously developed sol–gel procedure based on the co-hydrolysis and co-gelation of the metal and silica precursors as detailed in the Supplementary Materials [69,72].

Briefly, an ethanolic solution of TEOS is first pre-hydrolyzed under acidic catalysis by addition of an HCl hydroethanolic solution at 50 °C for 0.5 h. At this step, an ethanolic solution of metal precursors in stoichiometric amounts (divalent metal M precursor: Fe(III) precursor 1:2 molar ratio) is added under stirring. Basic catalysis is then promoted by the addition of an ethanolic solution of urea and kept under reflux for 2 h. Finally, the viscous sol is transferred in a container, sealed, and kept at 40 °C for gelation (20 to 40 h).

Aerogels are produced by high-temperature supercritical drying performed in an autoclave (Parr, Moline, IL, USA, 300 cm^3^) filled with 70 mL of absolute ethanol and nitrogen and brought to around 330 °C and 70 atm, and finally vented and cooled down to room temperature. To obtain the MFe_2_O_4_/SiO_2_ magnetic nanocomposites, the aerogels are first treated at 450 °C for 1 h in static air to remove synthesis residues and finally calcined in air at 900 °C for 1 h to promote the formation of nanocrystalline ferrite. In the case of pure iron oxide and of manganese ferrite, thermal treatment is performed under Ar flow (56 mL∙min^−1^) to avoid further oxidation.

### 3.3. Catalytic Tests

The reaction was carried out starting from stoichiometric amounts of reactants (77.9 mg of **1** and 50.3 mg of **2**) in 2 mL CH_2_Cl_2_ at room temperature under stirring in a closed flask. The catalytic effect of the aerogel was tested by adding a 5 mol% amount to the reaction mixture. Reaction time was 1 week, after which 2 mL of CH_2_Cl_2_ was added and the catalyst was separated from the reaction mixture by magnetic separation. Briefly, a magnet was placed on the outer side of the flask containing the reaction mixture. The aerogel catalyst was readily attracted by the magnet and the reaction mixture was collected. Fresh CH_2_Cl_2_ was then added and the magnetic separation procedure was repeated. The crude was separated by evaporation of CH_2_Cl_2_ under reduced pressure under rotavapor. In the case of non-magnetic catalyst (silica), the catalyst was separated from the reaction mixture by filtration and further processed as described above.

In view of the characterization of the aerogel after use, as well as for further catalyst reuse, after recovery and washing as described above, aerogel drying at around 50 °C in static air was performed.

### 3.4. Characterization Techniques

^1^H NMR spectra were recorded on a Varian 500 spectrometer (Palo Alto, CA, USA) at ambient temperature with CDCl_3_ as solvent. Data are reported as follows: chemical shifts (δ), multiplicity, coupling constants, and integration. ^13^C NMR spectra were recorded operating at 126 MHz at 27 °C with CDCl_3_ as solvent.

Textural characterization was performed on a Micromeritics ASAP2020 (Norcross, GA, USA) by determining N_2_ physisorption curves at −196 °C, after degassing the samples at 200 °C for 12 h. Surface areas were estimated using the Brunauer–Emmett–Teller (BET) model, whereas pore size and pore volumes were estimated by using the Barret–Joyner–Halenda (BJH) method.

Tian–Calvet heat flow equipment (Setaram, Lyon, France) was used for microcalorimetric measurements. Each sample was pretreated overnight at 400 °C under vacuum (10^−3^ Pa) before introducing ammonia as the probe gas for acidity. The adsorption temperature was maintained at 80 °C, in order to prevent physisorption. The equilibrium pressure relative to each adsorbed amount was measured by means of a differential pressure gauge (Datametrics, Plymouth, MN, USA). The run was stopped at a final equilibrium pressure of 133.3 Pa.

Scanning electron microscopy (SEM) investigation was performed on an ESEM FEI Quanta 200 microscope (Hillsboro, OR, USA) operating at 25 KV and on a ZEISS microscope (Jena, Germany). The powdered samples were deposited on conductive carbon tape for observation.

TEM images were recorded on a Hitachi H-7000 (Tokyo, Japan) and on a JEM 1400 Plus instrument (JEOL, Akishima, Japan) running at 100 kV. Prior to observation, the samples were finely ground and deposited on a carbon-coated copper grid.

XRD patterns were acquired on a Panalytical Empyrean diffractometer (Malvern Panalytical, Malvern, UK) equipped with Cu Kα radiation, a graphite monochromator on the diffracted beam, and an X’Celerator linear detector (Malvern Panalytical). The average size of crystallite domains was calculated by using the Scherrer equation, by considering the full width at half-maximum of the diffraction peak corrected for instrumental broadening as determined by using a reference LaB_6_ sample. Phase identification was performed by comparison to the Powder Diffraction Files database. Additional measurements were recorded on an X3000 Seifert diffractometer (GE Measurement and Control, Billerica, MA, USA).

Fourier Transform infrared (FT-IR) spectra in the Mid-IR range (4000–400 cm^−1^) were recorded on a Bruker Tensor 27 spectrometer (Billerica, MA, USA) equipped with a Platinum-ATR accessory and a DTGS (deuterated triglycine sulfate) detector.

## 4. Discussion

Nanocomposites, where nanocrystalline magnetic nickel ferrite with crystal size around 10 nm is dispersed in porous silica aerogel, were tested for the catalytic synthesis of 3,3′-((4-nitrophenyl)methylene)bis(1*H*-indole) starting from indole and 4-nitro benzaldehyde. The NiFe_2_O_4_-SiO_2_ nanocomposite exhibits high porosity and the occurrence of nanocrystals evenly distributed in the porous texture, exhibiting therefore promising textural and structural features for application in catalysis. Indeed, although some challenges remain in the reaction mixture characterization, it can be inferred that the Ni-CAT aerogel shows high activity towards the formation of BIMs even at room temperature, whereas the formation of the desired bis-heterocyclic product under catalyst-free conditions is nearly negligible. The activity of the nanocomposite catalyst can be ascribed both to the highly porous and interconnected texture typical of aerogels, and to the acidic features of the material (which seems to be due both to the occurrence of surface silanols as well as to the metal cations in the nanocrystals). Mechanistically, it is reasonable to assume that a contribution to the catalytic activity of the porous silica-based aerogels is associated with interactions, including hydrogen bonding/protonation, which are expected to be able to activate the electrophilic carbonyl of **2** (towards nucleophilic attack of **1**) in the first step of the reaction, leading to intermediate **A** as well as to promotion of the subsequent condensation reaction between **A** and a second equivalent of **1** via **B** (S_N_1-type reaction, see Figure 1). To take into account the role of the catalyst composition in the overall acidity, microcalorimetric measurements were performed using ammonia as a probe molecule to determine the strength and number of acidic sites. Reporting (Figure S6) the differential heat of ammonia adsorption (*Q*_diff_) as a function of the number of adsorbing sites per gram of catalyst (*n*_A_), it was found that SiO_2_-CAT is less acidic than the NiFe_2_O_4_-SiO_2_ nanocomposite aerogel. The moderate acidity of silica as deduced by microcalorimetry, together with the observed catalytic activity, suggest the active role of the porous silica.

To corroborate this finding, we have compared the catalytic activity of the NiFe_2_O_4_-SiO_2_ aerogel catalyst to the corresponding dry gel obtained by conventional thermal drying techniques (xerogel). In particular, NiFe_2_O_4_-SiO_2_ composites were prepared in the form of xerogel (Ni-X-CAT) and compared to the corresponding aerogel discussed above; while the structural features as obtained by XRD pattern suggest a similar crystal structure, the textural features as obtained by TEM and N_2_ physisorption suggest a significantly different texture (Figure S7). In particular, Ni-X-CAT exhibits a mainly microporous and fine texture, as compared to the meso- and macroporous morphology of the aerogel. Table 3 shows that, despite the same composition, NiFe_2_O_4_-SiO_2_ nanocomposites exhibit a different catalytic behavior in the forms of aerogel and of xerogel, suggesting a relevant role for the textural features and a better performance for the aerogel catalysts. Collectively, these observations suggest that both the acidity due to silanols and the typical texture of porous silica aerogels are believed to contribute to the catalytic activity of these materials and may be responsible for the observed results.

In conclusion, our preliminary study investigates the potential of nanocomposite aerogels in the catalytic production of relevant organic compounds, as demonstrated for the synthesis of bis(indolyl)methanes. NiFe_2_O_4_-SiO_2_ aerogels, as a consequence of their acidity combined with the typical texture of porous silica aerogels, enable the efficient synthesis of BIMs at room temperature. The high porosity and hydrophylic features of the silica matrix can also be responsible for the trapping of some species (including the intermediate or final product) involved during the reaction. The nanocrystalline ferrite provides magnetic properties for catalyst reusability in the development of cost-effective aerogel catalysts for the production of important heterocyclic compounds.

## Figures and Tables

**Table 1:** List of aerogel catalysts and corresponding relevant textural features. Thermal treatment was performed under static air atmosphere.

Aerogel Label	Processing Conditions	Composition (10 wt% Loading MFe_2_O_4_)	Surface Area (m^2^∙g^−1^)	Pore Volume (cm^3^∙g^−1^)
Ni-CAT	900 °C 1 h	NiFe_2_O_4_/SiO_2_	405	2.09
SiO_2_-CAT	900 °C 1 h	SiO_2_	373	1.36

**Table 2:** Catalytic tests.

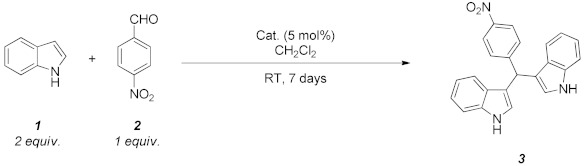		
Catalyst	1:2:3 Ratio ^1^	2:3 Ratio ^1^
None	44:48:8	86:14
SiO_2_	7:37:56	40:60
NiFe_2_O_4_-SiO_2_	4:2:94	2:98

^1^ All ratios were calculated by crude ^1^H NMR analysis and are normalized by the reaction stoichiometry.

**Table 3:** BIM synthesis ^1^ catalyzed by NiFe_2_O_4_-SiO_2_ aerogel (Ni-CAT) and xerogel (Ni-X-CAT) nanocomposites (catalyst amount: 5 mol%; run time: 1 week; solvent: CH_2_Cl_2_; reaction temperature: RT).

Catalyst (5 mol%)	1:2:3 Ratio ^1^	2:3 Ratio
Ni-X-CAT	20:34:46	43:57
Ni-CAT	4:2:94	2:98

^1^ All ratios were calculated by crude ^1^H NMR analysis.

## Data Availability

Data are contained within the article and Supplementary Materials.

## Supplementary Materials

The following supporting information can be downloaded at: https://www.mdpi.com/article/10.3390/molecules29102223/s1, Figure S1: XRD pattern of the NiFe_2_O_4_/SiO_2_ nanocomposite aerogel catalyst; Figure S2: N_2_ physisorption isotherms at 77 K of the NiFe_2_O_4_/SiO_2_ and SiO_2_ aerogel catalysts; Figure S3: ^1^H NMR and FT-IR spectrum of pure compound **3**; Figure S4.1–16: ^1^H NMR spectrum of crude reaction mixture under the different conditions tested; Table S1: Effect of solvent on the catalytic test; Table S2: Effect of catalyst reuse; Figure S5-1: TEM and SEM investigation of the Ni-CAT aerogel after catalysis; Figure S5-2: XRD pattern of the Ni-CAT aerogel after 3 runs of catalysis; Figure S6: Differential heat of adsorption of ammonia by the aerogels; Figure S7: TEM images, XRD pattern, and N_2_ physisorption isotherm at 77 K of the NiFe_2_O_4_/SiO_2_ nanocomposite xerogel catalyst (X-Ni-CAT).
