# Supplementary material for: Magnetic Aerogels for Room-Temperature Catalytic Production of Bis(indolyl)methane Derivatives
Source: Molecules. 2024 May 9;29(10):2223. doi: 10.3390/molecules29102223 (PMC11124404; doi:10.3390/molecules29102223)
Supplement: Supplementary file 1 [file molecules-29-02223-s001.zip › molecules-2966846-supplementary.pdf]

Supporting Materials for Manuscript

Magnetic Aerogels for Room Temperature Catalytic Production of Bis(indolyl)methane derivatives

Nicola Melis [1], Danilo Loche [2], Swapneel V. Thakkar [3,#], Maria Giorgia Cutrufello [3], Maria Franca Sini [3], Gianmarco Sedde [3], Luca Pilia [1], Angelo Frongia [3,*], and Maria Francesca Casula [1,*]

[1] Department of Mechanical, Chemical and Materials Engineering, University of Cagliari, I-09123 Cagliari, Italy

[2] King Abdullah University of Science and Technology, Biological and Environmental Science and Engineering (BESE) Division, NABLA Lab, 23955-6900 Thuwal, Saudi Arabia

[3] Department of Chemical and Geological Sciences, University of Cagliari, I-09042 Monserrato (Ca), Italy

[#] current affiliation: Tronox, Research Center Thann,95 Rue du Général de Gaulle, 68800 Thann, France

* Correspondence: mariaf.casula@unica.it (M.F.C.); afrongia@unica.it (A. F.)

S1. Preparation of Aerogel Catalysts

The aerogel catalysts were prepared based on previous protocols developed in our laboratories on the synthesis of silica-based nanocomposite aerogels. The protocol relies on the two step acid-base catalytic production of a multicomponent alcogel, followed by supercritical drying and reactive thermal treatments, as outlined in the experimental. The detailed recipe for a representative aerogel catalyst, namely Ni-CAT, corresponding to a composite made out of nanocrystalline $NiFe_2O_4$ dispersed on amorphous SiO2 with an overall ferrite loading of 10 wt%, is reported below.

A solution of 7.9 mL of TEOS (tetraethoxysilane, $Si(OC_2H_5)_4$, Aldrich 98%) in 3.0 mL of absolute ethanol was hydrolyzed by adding 4 mL of the acidic solution prepared as follows: 2 mL of nitric acid (HNO_3, Carlo Erba, 65%), 80 mL of absolute ethanol and 130 mL of distilled water. The so-prepared solution is heated at 50 °C for 30 minutes; once cooled to room temperature, an ethanolic solution of 0.8151 g of iron (III) nitrate ($Fe(NO_3)_3·9H_2O$, Aldrich, 98%) and 0.2875 g of nickel (II) nitrate ($Ni(NO_3)_2·6H_2O$ Aldrich, 100%) in 7.5 mL of ethanol was added.

A gelifying solution of urea (3.5130 g of $CO(NH_2)_2$, Sigma-Aldrich, >99.0%, in 5.0 mL of distilled water and 9.0 mL of absolute ethanol), was added to the sol. After heating at 85 °C for about 2 h, the sol was transferred into closed vials and kept at 40 °C until gelation.

The alcogels were submitted to high temperature supercritical drying in an autoclave in order to obtain very porous aerogels nanocomposites.

S2. Phase identification in the Aerogel Catalysts

Phase identification in the aerogel catalysts was performed by X-ray powder diffraction followed by comparison with the reference Powder Diffraction Files database [PDF-2 File, ICDD International Centre for Diffraction Data, 1601 Park Lane, Swarthmore, USA]. The catalyst shows the occurrence of the broad halo centered at 2θ ~ 22 which is due to the amorphous SiO_2 aerogel

matrix, and of broad peaks which can be ascribed to the corresponding mixed ferrite spinel phase, as reported in Figure S1. The *necklace* network of amorphous silica typical of aerogels is also observed in the TEM image reported in Figure S1, where darker spots associated to the nickel ferrite nanoparticles are also observed. The porous silica matrix hence acts as a support and provides a means to disperse nickel ferrite nanoparticles.

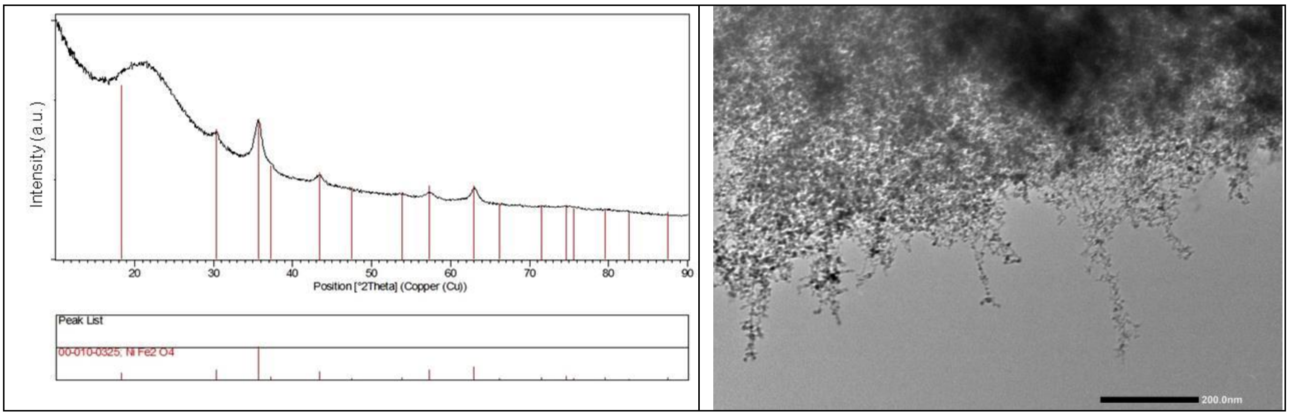

Figure S1. Left panel: XRD pattern of the $NiFe_2O_4/SiO_2$ nanocomposite aerogel catalyst showing peaks of nanosized ferrite on top of the halo of amorphous silica, and reference PDF card for the corresponding nanocrystalline phase. Data Analysis was performed by X'Pert High Pro Panalytical Software and phase identification were based on the Powder Diffraction Files. Right panel: TEM image of the $NiFe_2O_4/SiO_2$ nanocomposite aerogel catalyst.

S2. Textural characterization of the Aerogel Catalysts

The Ni-CAT aerogel catalyst has a texture which is mainly dictated by the silica matrix. Figure S2 shows representative N_2 adsorption-desorption isotherm at 77K for the Ni-CAT and for the SiO_2 aerogel catalyst.

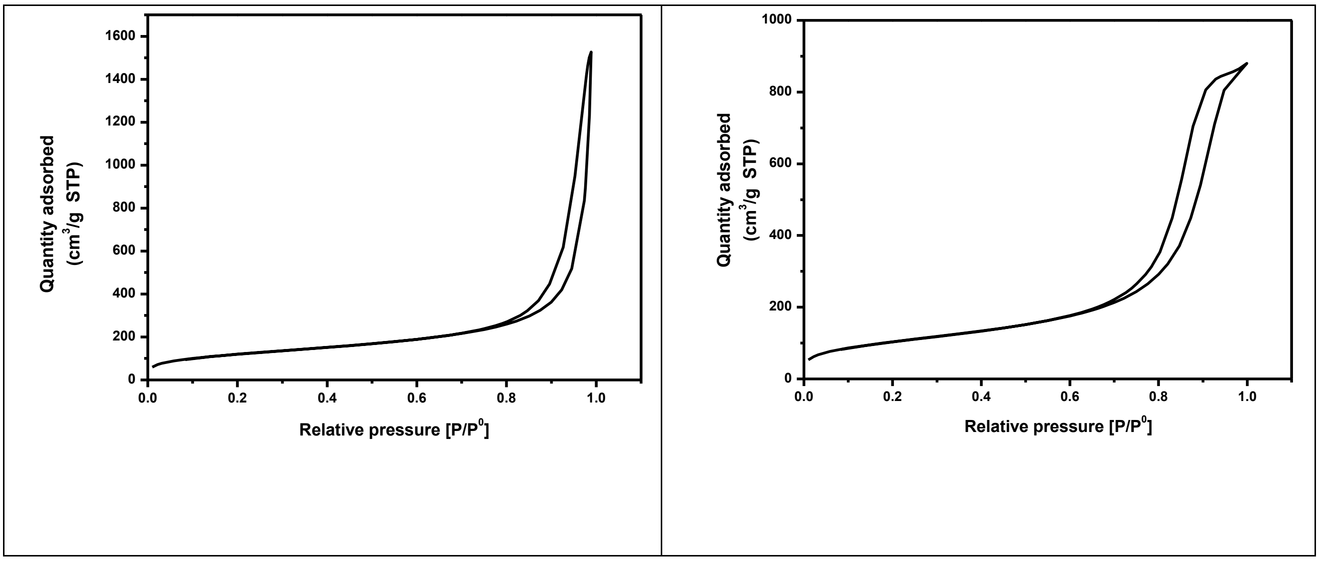

Figure S2. N_2 physisorption isotherms at 77 K of the $NiFe_2O_4/SiO_2$ (left) and SiO_2 (right) aerogel catalysts.

S3. Characterization of final compound 3

General Methods – ^{1}H NMR spectra were recorded on a Varian 500 spectrometer at ambient temperature with $CDCl_3$ as solvent. Data are reported as follows: chemical shifts (δ), multiplicity, coupling constants and integration. 13C NMR spectra were recorded operating at 126 MHz at 27 °C with $CDCl_3$ as solvent. FTIR spectra were recorded on a Bruker Tensor 27 spectrometer equipped with a Platinum-ATR accessory and a DTGS (deuterated triglycine sulfate) detector.

General Synthesis of compound 3 — 50.3 mg of *p*-nitrobenzaldehyde (0.333 mmol, 1 equiv.) and 77.9 mg of indole (0.666 mmol, 2 equiv.) were dissolved in 2 mL of solvent in a close vial with the aerogel catalyst (5%mol of the ferrite phase) and stirred for seven days. Reactions were therefore diluted with DCM and the catalyst was removed by means of a magnet followed by several washes. The filtrate was then concentrated under reduced pressure. Precipitation of pure compound 3 can be accomplished with the addition of water to an ethanol solution. A small portion of the filtrate was concentrated separately for NMR analysis in $CDCl_3$. When the reaction was tested with no catalyst, the crude mixture was diluted in order to solubilize all the products and, after a representative portion for NMR was taken, was concentrated under reduced pressure. When the reaction was tested with plain silica, the crude mixture was filtered and concentrated under reduced pressure.

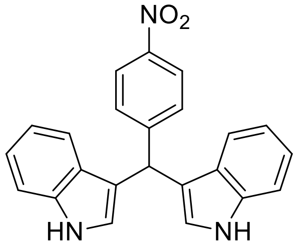

3,3'-((4-nitrophenyl)methylene)bis(1H-indole) (**3**) – Yellow solid. IR: 3458, 3424, 3385, 1592, 1505, 1458, 1338, 1014, 810, 792, 742 cm^{-1}. ^{1}H NMR (500 MHz, $CDCl_3$) δ 8.14 (d, *J* = 8.2 Hz, 2H), 8.01 (s, 2H), 7.51 (d, *J* = 8.3 Hz, 2H), 7.39 (d, *J* = 8.2 Hz, 2H), 7.34 (d, *J* = 8.0 Hz, 2H), 7.20 (t, *J* = 7.6 Hz, 2H), 7.03 (t, *J* = 7.5 Hz, 2H), 6.69 (s, 2H), 6.00 (s, 1H). ^{13}C NMR (126 MHz, $CDCl_3$) δ 155.58, 136.85, 133.16, 129.67, 126.81, 123.81, 123.77, 122.51, 120.28, 119.77, 119.71, 118.30, 111.40.

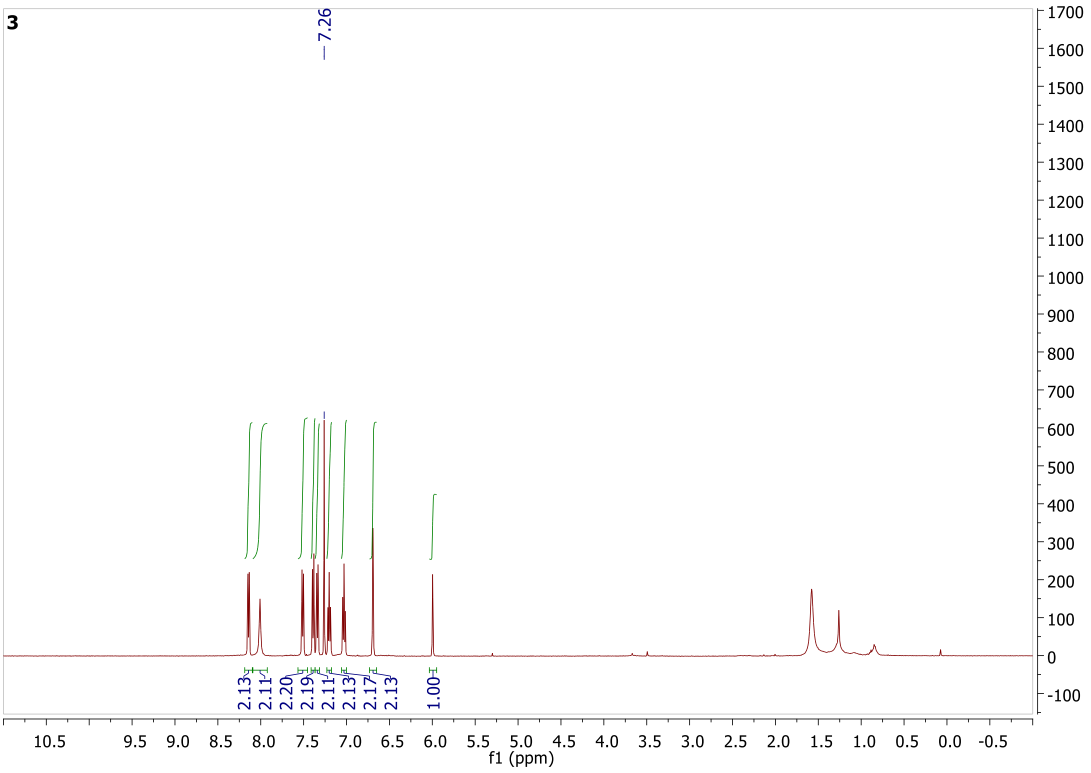

Figure S3-1. ^{1}H NMR spectrum of pure compound 3.

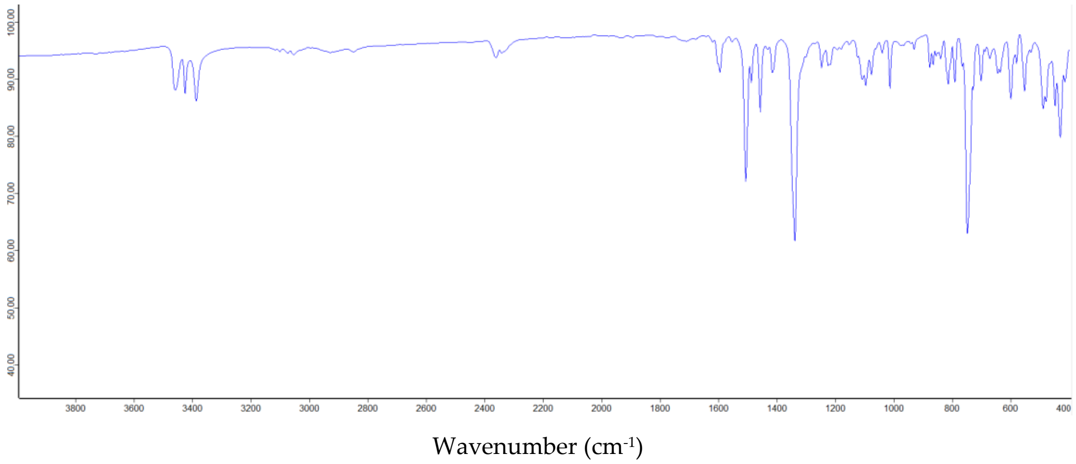

Figure S3-2. FT-IR spectrum of pure compound 3.

S4. ^{1}H NMR spectroscopy investigation of the reaction

^{1}H NMR analysis of the crude mixtures was used for determining the rough ratios between *p*-nitrobenzaldehyde (CHO signal at 10.4 ppm), indole (proton in 2 position at 6.5 ppm), and the final product **3** (CH alyphatic proton at 6.0 ppm). Final ratios take into account the stoichiometry of the reaction by considering half of indole intensity.

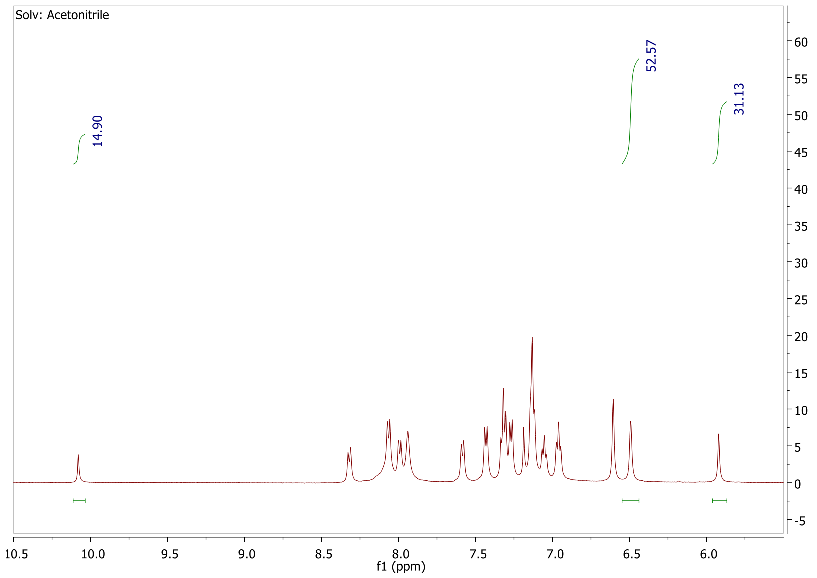

Figure S4-1. ^{1}H NMR spectrum of crude reaction mixture in acetonitrile with $NiFe_2O_4@SiO_2$ as catalyst.

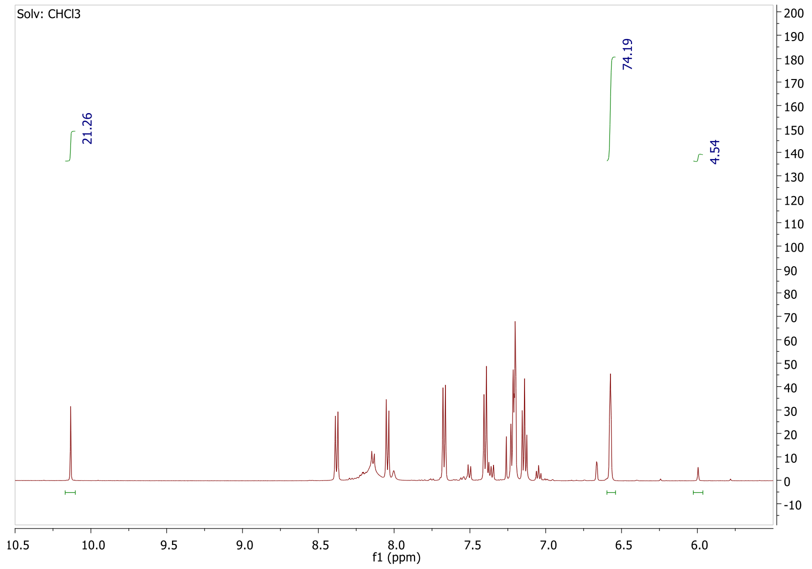

Figure S4-2. ^{1}H NMR spectrum of crude reaction mixture in chloroform with $NiFe_2O_4@SiO_2$ as catalyst.

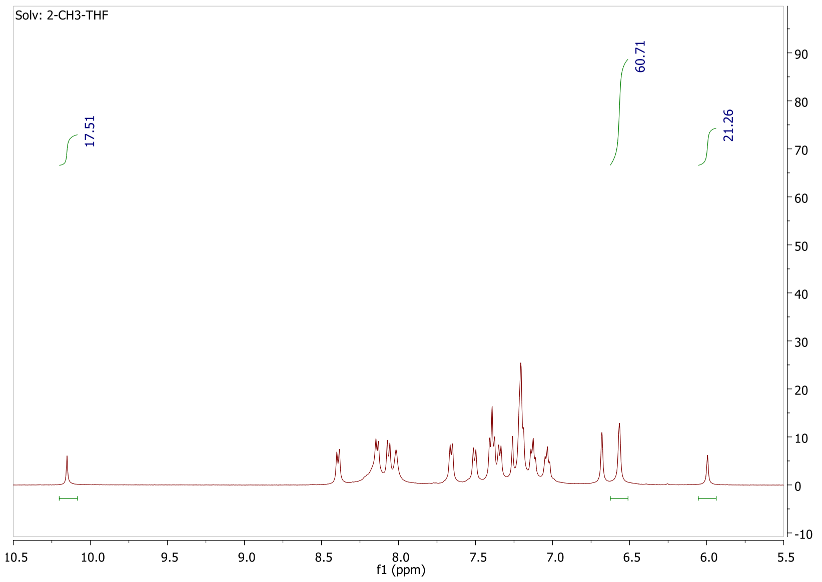

Figure S4-3. ^{1}H NMR spectrum of crude reaction mixture in 2-methy-THF with $NiFe_2O_4@SiO_2$ as catalyst.

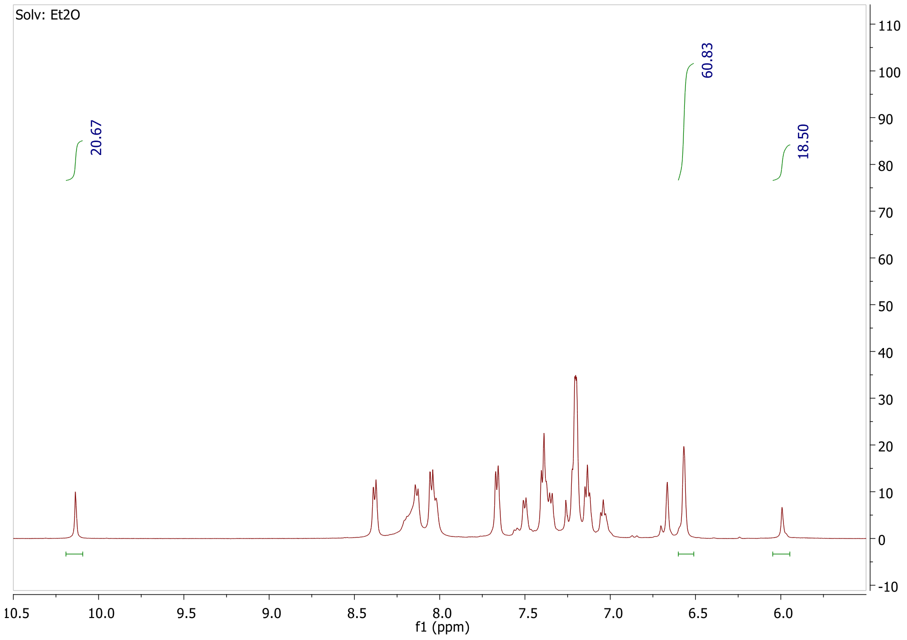

Figure S4-4. ^{1}H NMR spectrum of crude reaction mixture in diethyl ether with $NiFe_2O_4@SiO_2$ as catalyst.

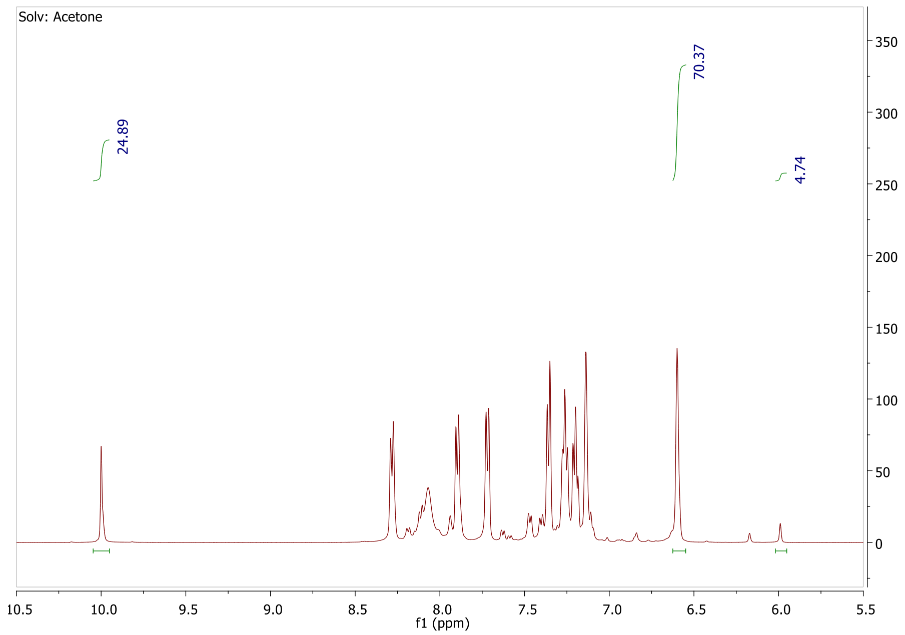

Figure S4-5. ^{1}H NMR spectrum of crude reaction mixture in acetone with $NiFe_2O_4@SiO_2$ as catalyst.

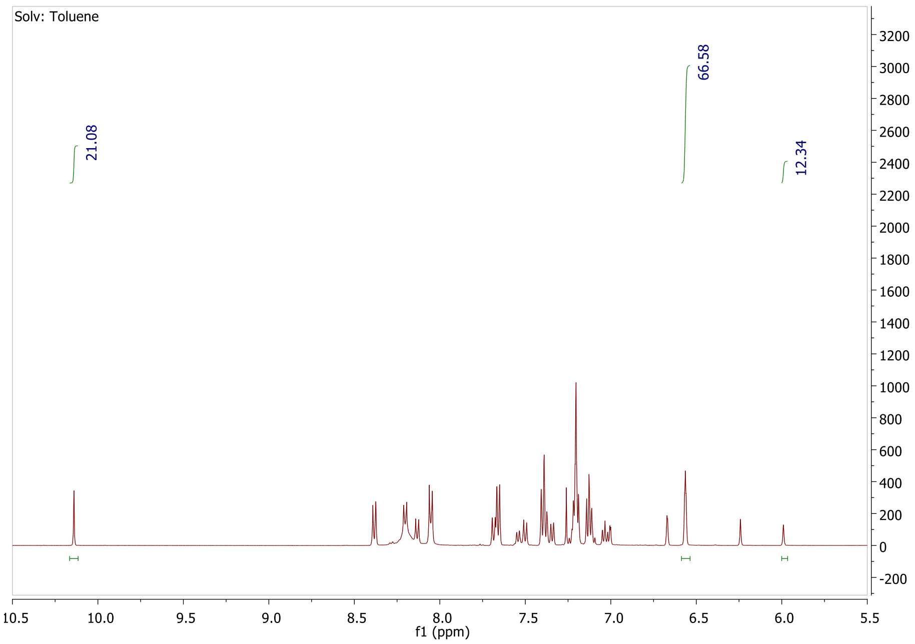

Figure S4-6. ^{1}H NMR spectrum of crude reaction mixture in toluene with $NiFe_2O_4@SiO_2$ as catalyst.

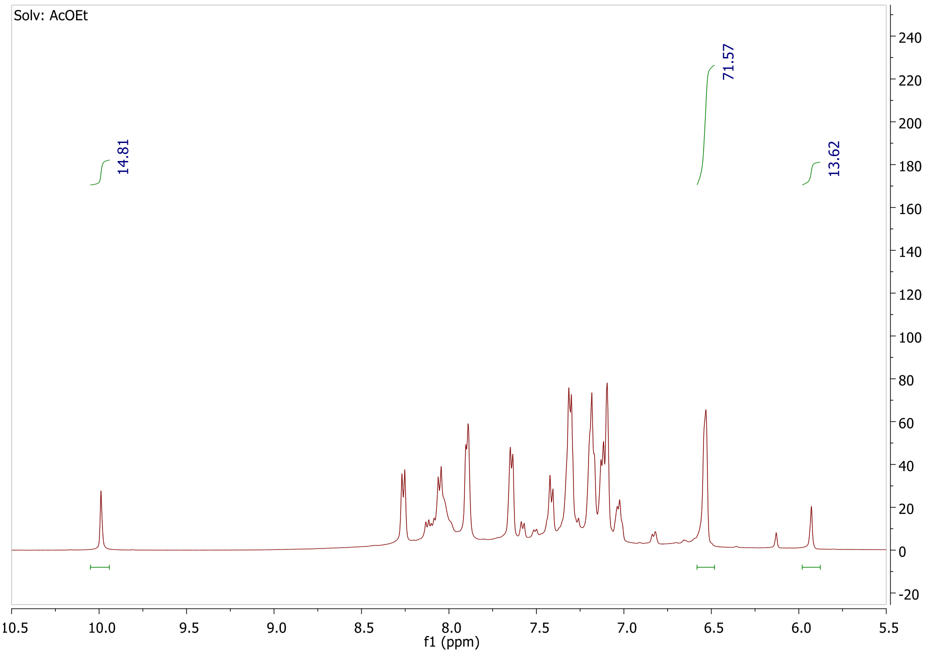

Figure S4-7. ^{1}H NMR spectrum of crude reaction mixture in ethyl acetate with $NiFe_2O_4@SiO_2$ as catalyst.

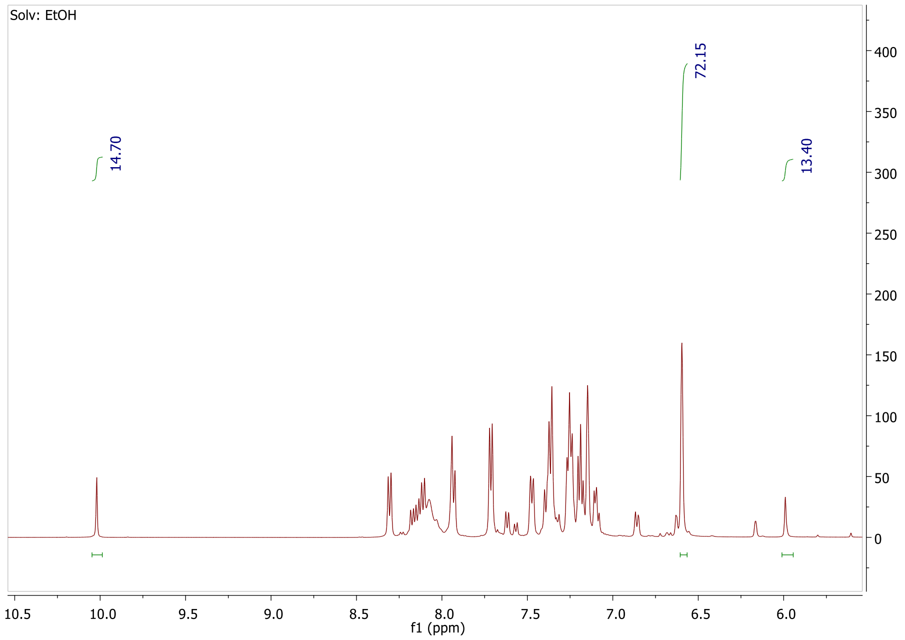

Figure S4-8. ^{1}H NMR spectrum of crude reaction mixture in ethanol with $NiFe_2O_4@SiO_2$ as catalyst.

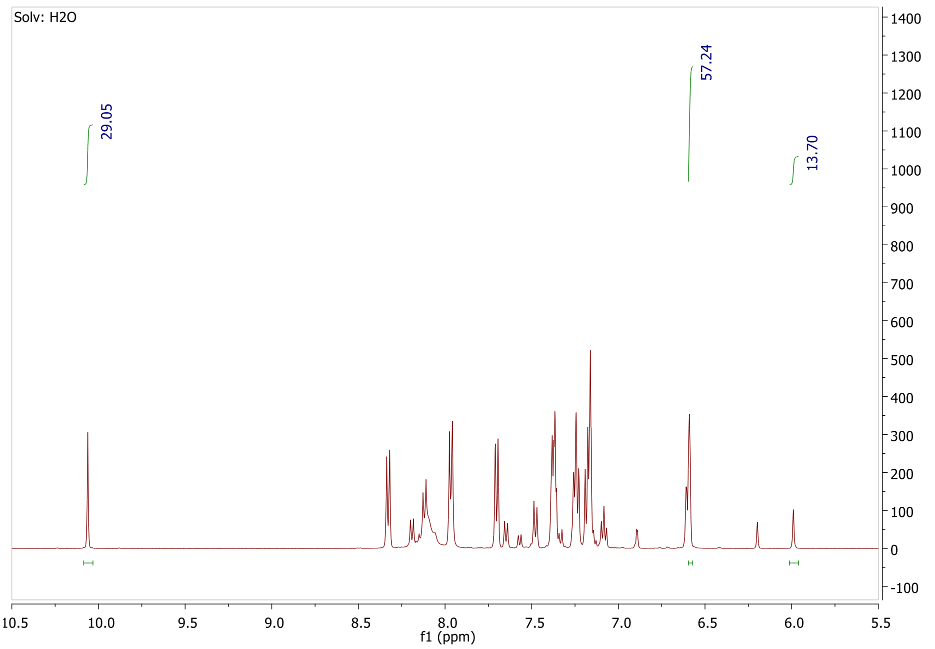

Figure S4-9. ^{1}H NMR spectrum of crude reaction mixture in acetonitrile with $NiFe_2O_4@SiO_2$ as catalyst.

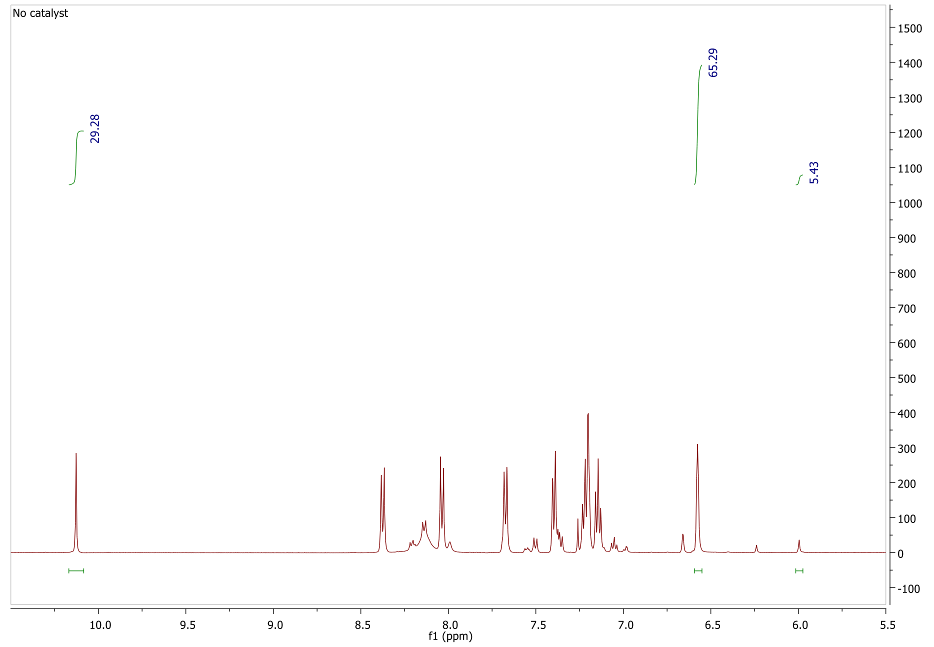

Figure S4-10. ^{1}H NMR spectrum of crude reaction mixture in DCM with no catalyst.

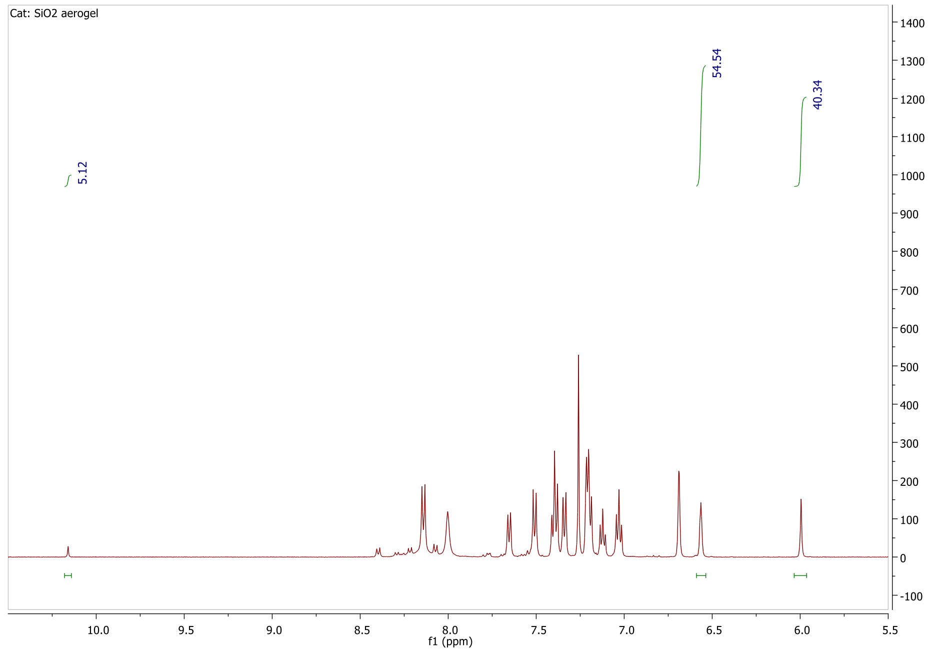

Figure S4-11. ^{1}H NMR spectrum of crude reaction mixture in DCM with plain silica aerogel as catalyst.

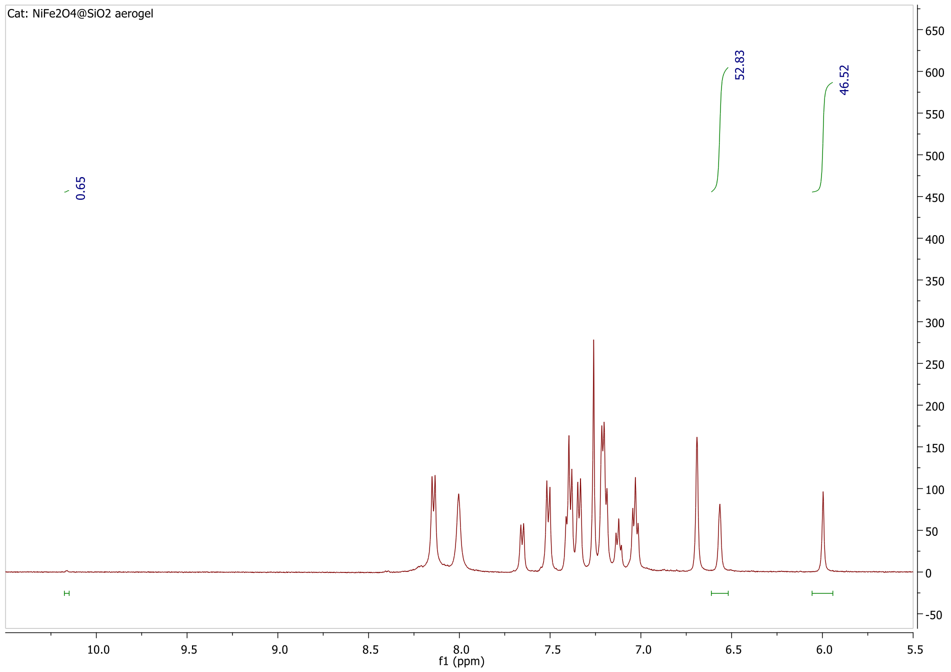

Figure S4-12. ^{1}H NMR spectrum of crude reaction mixture in DCM with $NiFe_2O_4@SiO_2$ as catalyst.

.

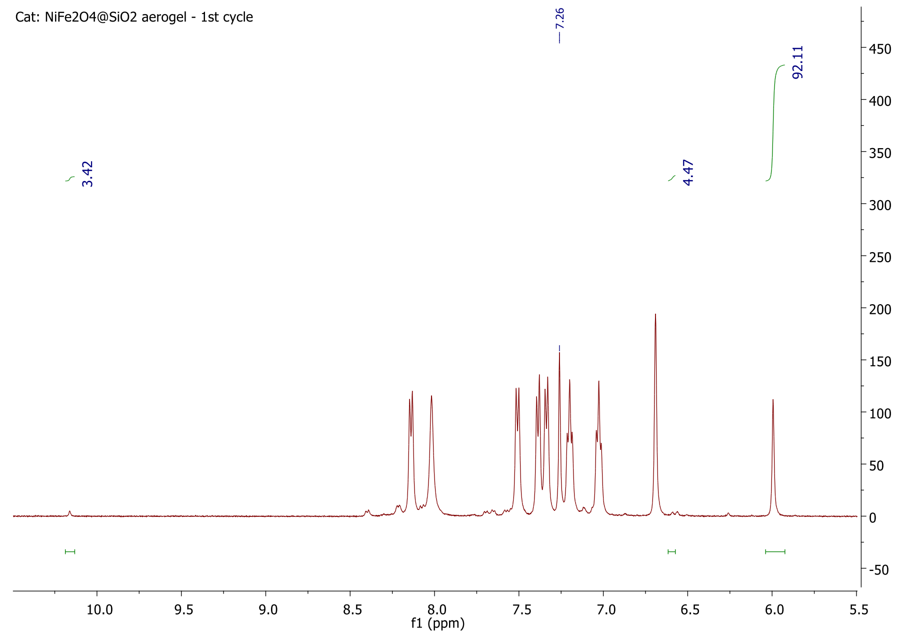

Figure S4-13. ^{1}H NMR spectrum of crude reaction mixture in DCM with $NiFe_2O_4@SiO_2$ as catalyst at the first cycle of three.

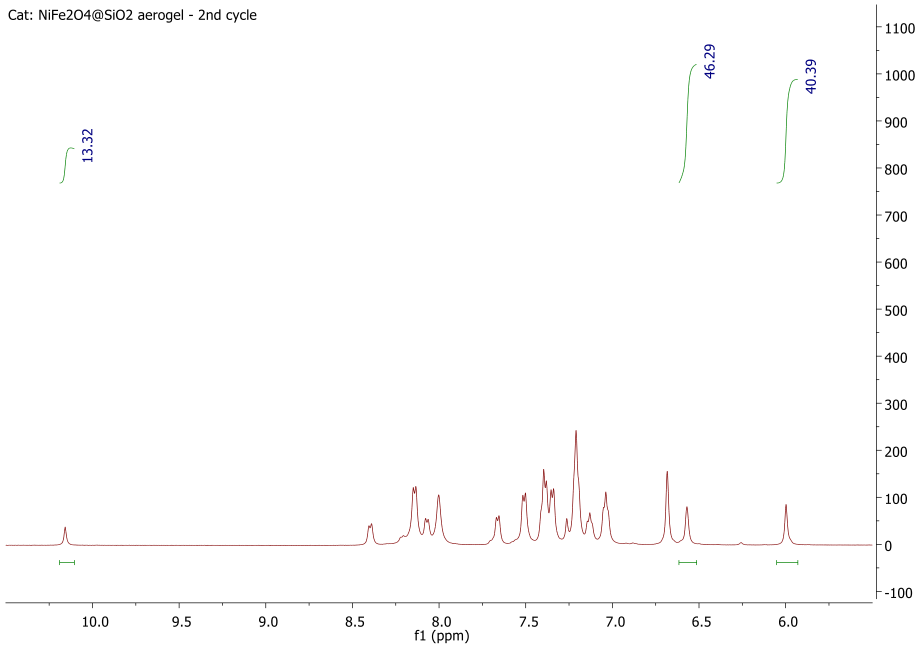

Figure S4-14. ^{1}H NMR spectrum of crude reaction mixture in DCM with $NiFe_2O_4@SiO_2$ as catalyst at the second cycle of three.

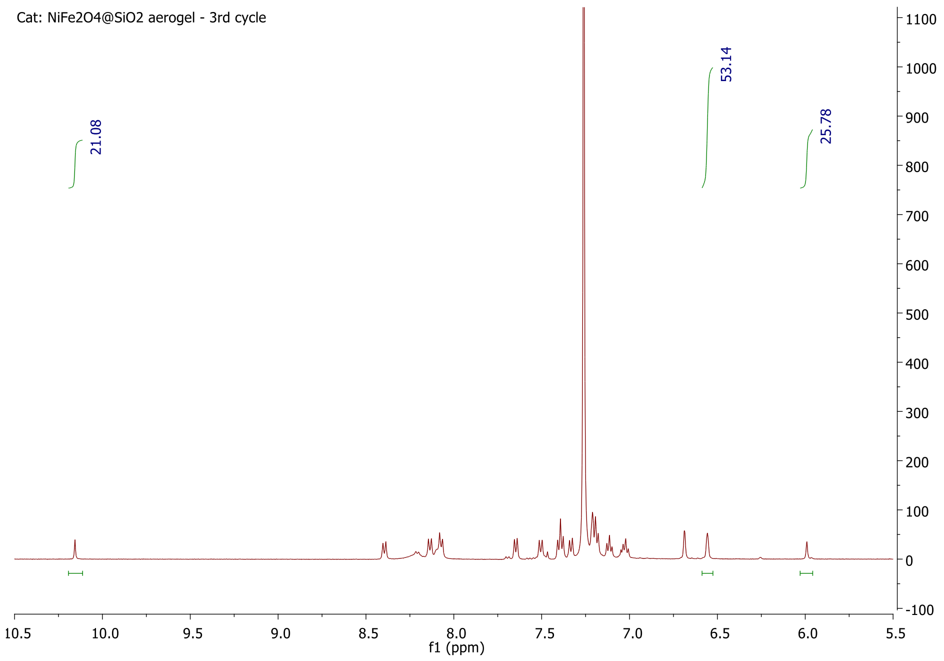

Figure S4-15. ^{1}H NMR spectrum of crude reaction mixture in DCM with $NiFe_2O_4@SiO_2$ as catalyst at the third cycle of three.

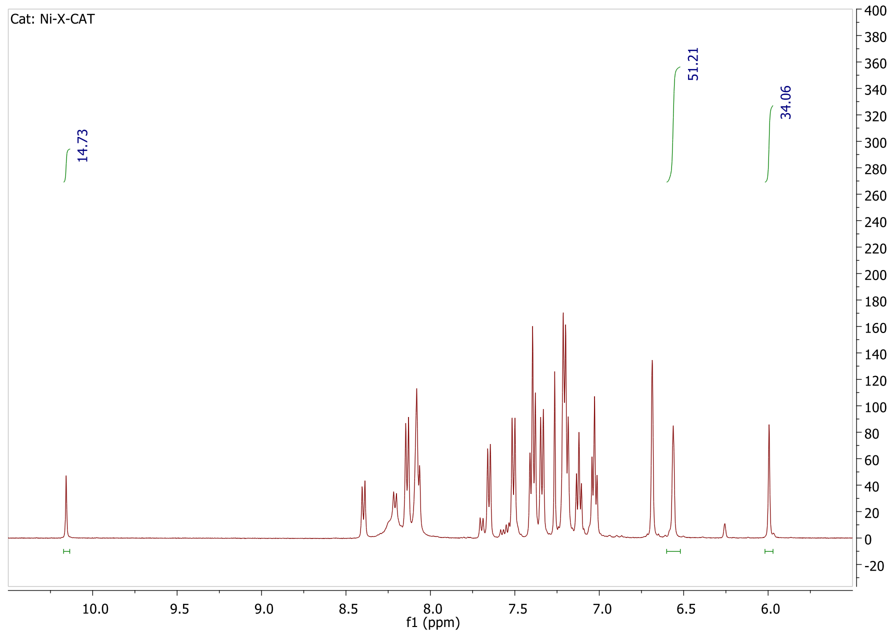

Figure S4-16. ^{1}H NMR spectrum of crude reaction mixture in DCM with Ni-AERO as catalyst.

S5. Solvent and Reuse Experiments.

The catalytic synthesis of BIMs in the presence of Ni-CAT in different solvents was performed. As shown in Table S1, the results suggest a strong dependence of the reaction on the nature of the solvent, which can be related both to the polarity, viscosity or coordinating properties of the solvent, as well as to the stability of the porous texture of the aerogels in different media. It was found that CH_2Cl_2 provides the best results and was used therefore for all further experiments.

Table S1. Effect of solvent on the catalytic test (catalyst: Ni-CAT, catalyst amount: 5 mol %; run time: 1 week; reaction temperature: RT).

Solvent	1:2:3 ratio[1]	2:3 ratio
CH_3CN	21:36:43	46:54
CH_2Cl_2	4:2:94	2:98
CH_3Cl	34:59:7	89:11
2-CH_3THF	25:44:31	59:41
Et_2O	30:44:27	62:38
Acetone	38:56:7	88:12
Toluene	32:50:18	73:27
EtOH	23:56:21	73:27
H_2O	41:40:19	68:32

[1] All ratios were calculated by crude ^{1}H NMR analysis.

Magnetic separation was exploited to remove the aerogel magnetic catalyst from the product mixture. After washing and drying the aerogels were tested again, showing catalytic activity as shown in Table S2.

Table S2. Effect of catalyst reuse (catalyst amount: 5 mol %; run time: 1 week; reaction temperature: RT).

Catalyst (5 mol%)	1:2:3 ratio[1]	2:3 ratio
Ni-CAT-1st run	4:2:94	2:98
-2nd run	20:29:51	36:64
-3rd run	28:36:35	51:49

[1] All ratios were calculated by crude ^{1}H NMR analysis.

Characterization of the aerogels after catalysis did not show any significant change as compared to the fresh catalyst, as shown in Fig. S5-1 for the case of the Ni-CAT sample. In particular, TEM and SEM of the Ni-CAT sample after use for two and three catalytic runs show that the catalyst still exhibits the typical porous texture of aerogels and that the ferrite nanocrystals are dispersed within the silica matrix.

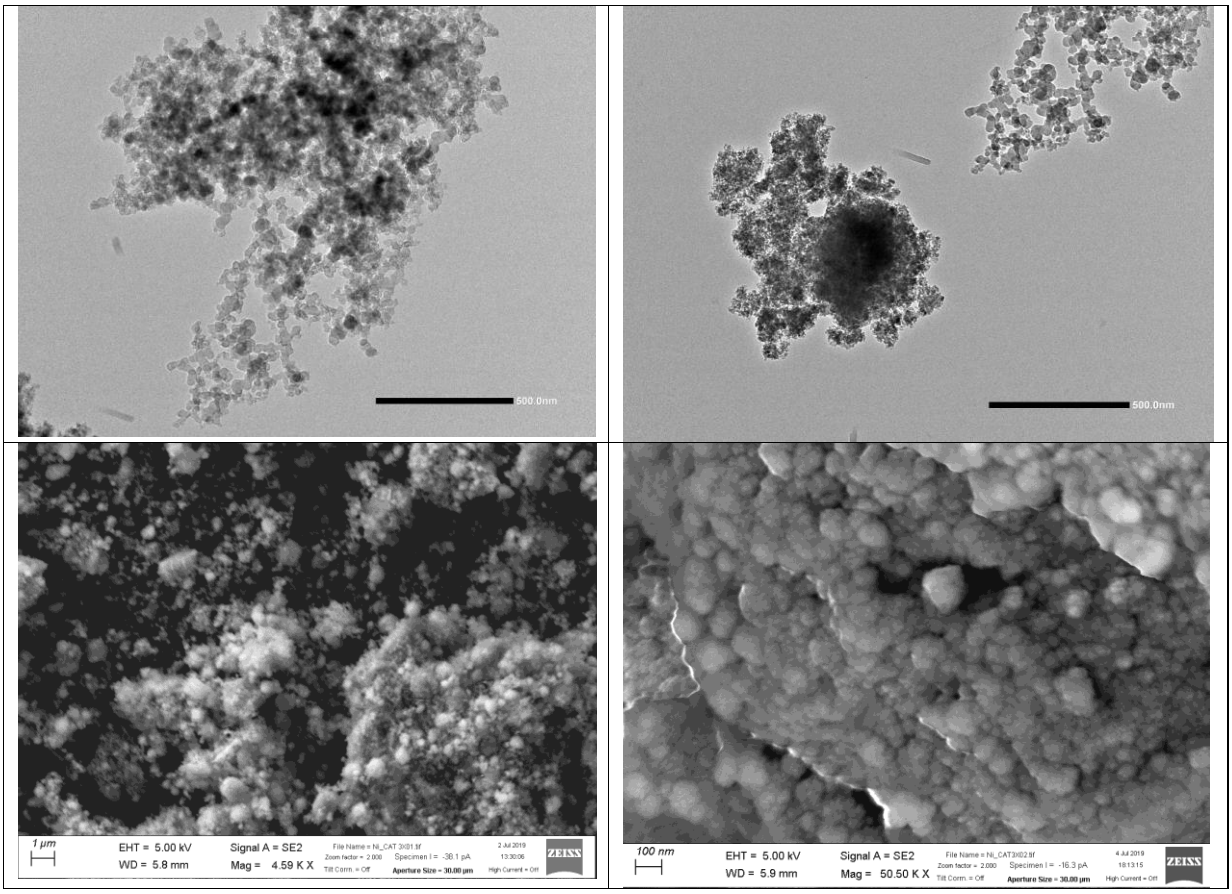

Figure S5-1. TEM (top) and SEM (bottom) investigation of the Ni-CAT aerogel after catalysis.

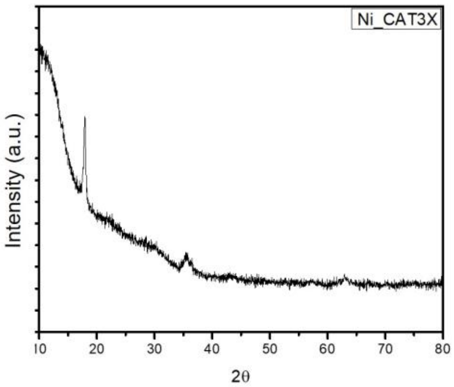

Figure S5-2. XRD pattern of the Ni-CAT aerogel after 3 runs of catalysis

S6. Microcalorimetric Characterization

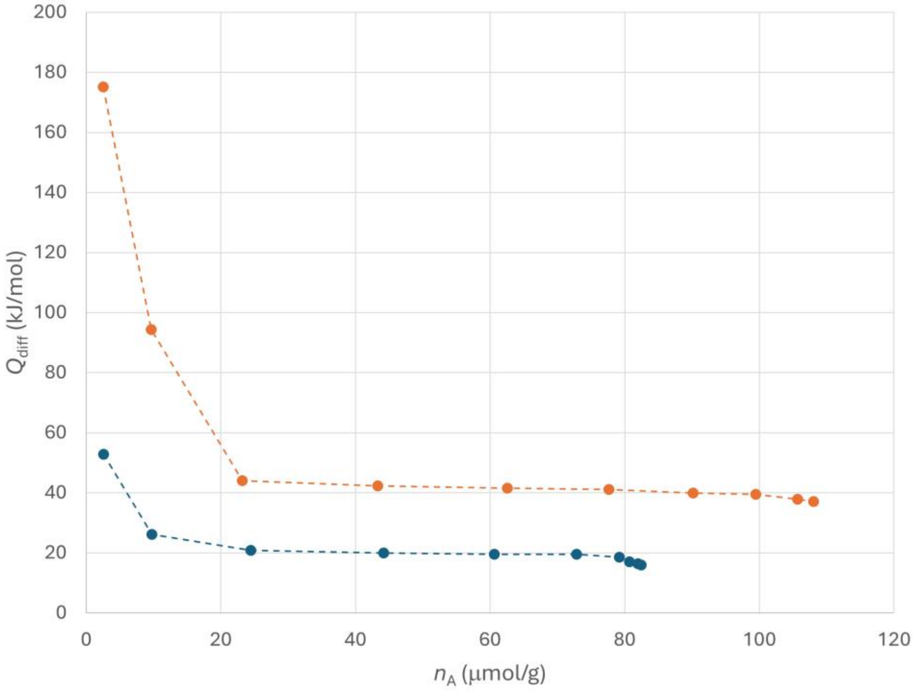

Figure S6. Differential heat of adsorption of ammonia as a function of the number of acid sites for plain silica (SiO_2-CAT, blue) and nickel ferrite-silica nanocomposite ($NiFe_2O_4$-SiO_2, red) aerogels.

S7. Characterization of the Xerogel Catalysts

Xerogel $NiFe_2O_4/SiO_2$ catalysts were prepared as a reference starting from the same alcogel followed by drying by conventional thermal treatments. The resulting nanocomposite xerogels have a relatively denser and microporous texture. Figure SI-6 shows representative TEM images, XRD and physisorption isotherms of X-Ni-CAT. As opposed to the aerogel samples (see Figure S2), the xerogel is microporous (relevant textural parameters for X-Ni-CAT are S_{BET}= 378 m^2/g and V_{pore}= 0.21 cm^3/g), hence the pore size is smaller and the average nanocrystal size of the formed nickel ferrite is smaller.

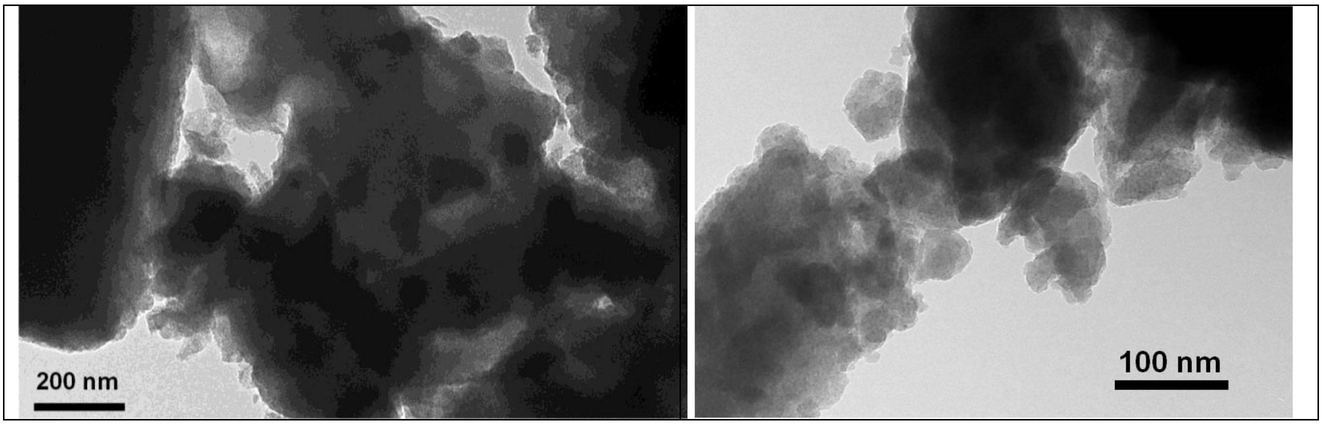

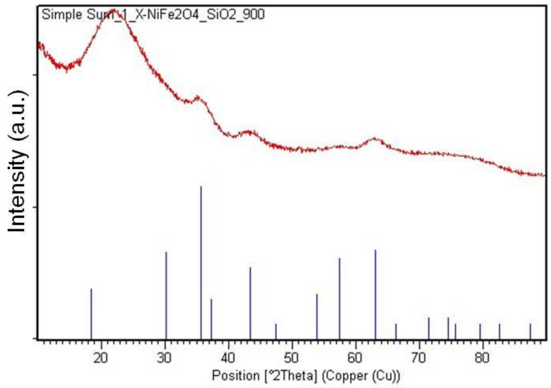

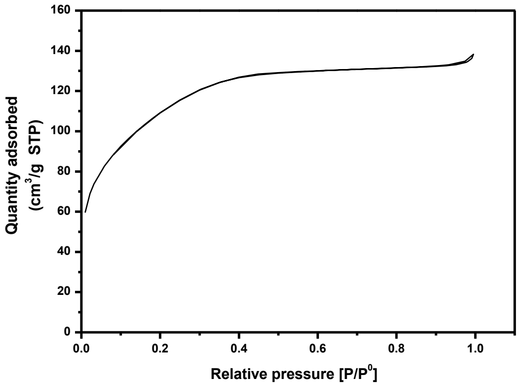

Figure S7. Representative TEM images, XRD pattern, and N_2 physisorption isotherm at 77 K of the $NiFe_2O_4/SiO_2$ nanocomposite xerogel catalyst (X-Ni-CAT)
